# Effectiveness of the Healthy Start-Départ Santé approach on physical activity, healthy eating and fundamental movement skills of preschoolers attending childcare centres: a randomized controlled trial

**DOI:** 10.1186/s12889-020-08621-9

**Published:** 2020-04-19

**Authors:** Anne Leis, Stéphanie Ward, Hassan Vatanparast, M. Louise Humbert, Amanda Froehlich Chow, Nazeem Muhajarine, Rachel Engler-Stringer, Mathieu Bélanger

**Affiliations:** 1 Department of Community Health & Epidemiology, College of Medicine, University of Saskatchewan, Health Sciences E Wing, 104, Clinic Place, Saskatoon, SK S7N 5E5 Canada; 2 École des sciences des aliments, de nutrition et d’études familiales, Faculté des sciences de la santé et des services communautaires, Université de Moncton, Moncton, Canada; 3 College of Pharmacy and Nutrition/School of Public Health, University of Saskatchewan, Saskatoon, Saskatchewan S7N 0Z2 Canada; 4 College of Kinesiology, University of Saskatchewan, Saskatoon, Canada; 5 Department of family medicine, Université de Sherbrooke, 18 avenue Antonine-Maillet Moncton, Moncton, New Brunswick E1A 3E9 Canada; 6 Centre de formation médicale du Nouveau-Brunswick, 18 avenue Antonine-Maillet Moncton, Moncton, New Brunswick E1A 3E9 Canada; 7 Vitalité Health Network, 330 Université Avenue Moncton, Moncton, New Brunswick E1C 2Z3 Canada

**Keywords:** Preschool, Physical activity, Eating behaviours, Food intake, Fundamental movement skills, Population health intervention

## Abstract

**Background:**

Since young children spend approximately 30 h per week in early childcare centres (ECC), this setting is ideal to foster healthy behaviours. This study aimed to assess the effectiveness of the Healthy Start-Départ Santé (HSDS) randomized controlled trial in increasing physical activity (PA) levels and improving healthy eating and fundamental movement skills in preschoolers attending ECC.

**Methods:**

Sixty-one ECC were randomly selected and allocated to either the usual practice (*n* = 30; *n* = 433 children) or intervention group (*n* = 31; *n* = 464 children). The HSDS intervention group was provided a 3-h on-site training for childcare educators which aimed to increase their knowledge and self-efficacy in promoting healthy eating, PA and development of fundamental movement skills in preschoolers. PA was measured during childcare hours for five consecutive days using the Actical accelerometer. Preschoolers’ fundamental movement skills were assessed using the standard TGMD-II protocol and POMP scores. Food intake was evaluated using digital photography-assisted weighted plate waste at lunch, over two consecutive days. All data were collected prior to the HSDS intervention and again 9 months later. Mixed-effect models were used to analyse the effectiveness of the HSDS intervention on all outcome measures.

**Results:**

Total number of children who provided valid data at baseline and endpoint for PA, food intake and fundamental movement skills were 259, 670 and 492, respectively. Children in the HSDS intervention group had, on average, a 3.33 greater point increase in their locomotor motor skills scores than children in the control group (β = 3.33, *p* = 0.009). No significant differences in effects were observed for object control, PA and food intake. However, results demonstrated a marginal increase in portions of fruits and vegetables served in the intervention group compared to control group (β = 0.06, *p* = 0.05).

**Conclusion:**

Of the 12 outcome variables investigated in this study, 10 were not different between the study groups and two of them (locomotor skills and vegetables and fruits servings) showed a significant improvement. This suggests that HSDS is an effective intervention for the promotion of some healthy behaviours among preschoolers attending ECC.

**Trial registration:**

Clinical Trials NCT02375490. Registered on February 24, 2015; 77 retrospectively registered.

## Background

It is well documented that early childhood (0–5 years) sets the foundation for a lifetime of health and well-being [1]. However, research indicates that very young Canadian children are not active enough [2] and may not have a sufficiently nutritionally balanced diet for optimal growth and development [3]. Given that young children in many developed countries spend approximately 30 h per week in early childcare centres (ECC), [4] this setting has been identified as an ideal environment for implementing strategies to foster the development of healthy behaviours [5, 6].

While much effort has already been invested in either improving physical activity or healthy eating among school age children and preschoolers, interventions have rarely assessed both behaviours simultaneously. A key aspect of increasing physical activity is the development of fundamental movement skills (e.g., object control and locomotor skills); to date this has often been overlooked. Several reviews have also highlighted the limited impact of single domain interventions [7, 8]. For example, one systematic review reports that the least successful interventions in improving physical activity levels, dietary behaviours, or body composition focused on only one or two outcomes; conversely, the most successful interventions aimed to positively influence several factors, such as knowledge, abilities and competence [9]. Accordingly, interventions should be grounded in comprehensive behaviour change models and include a multipronged approach [10]. Interventions promoting healthy weights in children should therefore, encompass a broad spectrum of concerted actions targeting both physical activity and healthy eating [6] and should be based on best available knowledge from research and practice [8, 11].

Built on a socioecological model, Healthy Start-Départ Santé (HSDS) was developed following the principles described above and includes strategies for each level of influence. HSDS is a multilevel, intersectoral population health intervention designed to empower childcare educators to enhance physical activity, fundamental movement skills and healthy eating opportunities within the daily routine of preschoolers (3 to 5 years old) who attend ECC (i.e., licenced childcare centres or preschools). HSDS adheres to the population health approach which posits that to positively influence population-level health outcomes, interventions must take into account the wide range of health determinants, [12] recognize the importance and complexity of potential interplay among these determinants, and reduce social and material inequities [13]. Further, they must rely on the best available evidence, stimulate intersectoral collaborations, and provide opportunities for all potential stakeholders to be meaningfully engaged from the onset to deployment [13].

The HSDS evaluation reported here aimed to assess the effectiveness of the HSDS intervention in increasing physical activity levels and healthy eating as well as improving fundamental movement skills in preschoolers attending ECC. It was hypothesized that, in comparison to a control group (usual practice), exposure to the HSDS intervention would result in increased opportunities for physical activity and healthy eating, which in turn would lead to increased physical activity levels, improved fundamental movement skills and healthier eating among preschoolers.

## Methods

### Trial design

This study used an ECC-based cluster randomized controlled trial design, where ECC were randomly allocated to either the intervention (HSDS) or control group (usual practice). A complete description of the trial protocol was published in 2016 and is registered (ClinicalTrials.gov #NCT02375490) [14]. The study protocol was implemented as planned; however, modifications were made in the method used to score fundamental movement skills as explained below. Further, as detailed in the analysis section, the amount of missing data for the outcomes forced us to modify the analysis plan from an intention-to-treat to a complete-cases analysis approach. The study received ethics approval from Health Canada, the University of Saskatchewan, and the Université de Sherbrooke.

### Participants

Provincial registries of licenced ECC in Saskatchewan and New Brunswick, Canada, were used as sampling frames. ECC were excluded if they had previously received a physical activity or nutrition intervention, did not prepare and provide lunch to children, and for feasibility reasons, if they had less than 20 children enrolled full-time in a preschool program. ECC were stratified according to province, geographical location (urban/rural) [15] and their respective school division (English or French). Once stratification was completed, project coordinators randomly selected ECC using the Stata SE statistical sequence generator software. ECC were then contacted, provided information about the study, and invited to participate. ECC which agreed to participate in the study were sent a consent form, as well as parental consent forms to recruit preschoolers attending their ECC on a full-time basis. If the ECC declined, they were replaced by another randomly selected ECC from the same stratum. Once informed consent was obtained, simple randomization was used to allocate ECC to either the intervention or control group with a 1:1 ratio. Parents of all participating children provided signed, informed consent. Prior to initiating recruitment and based on pilot work, we estimated that 700 children (350 per group) would provide 80% power to detect a 10% between-group difference in outcomes, considering a within-group standard deviation of 40%, a two-sided α of 0.05, an intra-class correlation of 0.02 and an estimated multiple correlation of 0.15 between the intervention and other explanatory variables. To compensate for losses to follow-up, our target was to recruit a minimum of 735 participants (5% over the 700 calculated).

### Intervention

The HSDS intervention was delivered over the course of 6 to 8 months, and included a 3-h on-site training, resources (i.e. an implementation manual, physical activity and healthy eating manuals, an active play equipment kit), and on-going on-line and telephone support and monitoring; centres were also offered a tailored 90-min booster session at the midway point of the intervention period. ECC randomly allocated to the control group continued their usual practice and were not provided with any training, resources or support. However, once the study was completed, all childcare centres from the control group were offered the HSDS intervention.

#### On-site training and resources

All ECC allocated to the intervention group were provided with a 3-h on-site training, which was offered to childcare educators, directors and cooks after work hours. This training session was delivered by trained specialists (dietitians, kinesiologists or other experts in the fields of nutrition and physical activity), and covered best practices in physical activity and healthy eating in early childhood, including topics such as the importance of physical activity and healthy eating for preschoolers, how to easily integrate physical activity and healthy eating in the ECC’s daily routine, how to introduce and encourage children to try new and healthy foods, and how to help children develop their fundamental movement skills. ECCs were also provided with the evidence-based LEAP BC™-GRANDIR CB resources which included a physical activity and healthy eating manual. In addition, a New Brunswick developed fundamental movement skills manual (Active Kids Toolkit Foundations for All©), a kit with active play equipment, an implementation manual, and other complementary resources for childcare staff and families were shared with all participating sites.

#### On-going support and monitoring

ECC were encouraged to identify a *Healthy Star*, which was a childcare staff member who was a champion for physical activity and healthy eating and who was a knowledge-sharing contact between the ECC and the HSDS coordinators. The HSDS team checked-in with the intervention ECC on a regular basis by phone or email and provided them with support and encouragement. Monthly newsletters were also sent to all intervention ECC, which included tips on how to get children moving or on how to improve healthy eating. ECC were encouraged to share these newsletters with parents.

#### Booster session

A 90-min booster session was offered to all intervention ECC approximately three months after the initial training. This on-site session was personalized based on challenges identified by each individual ECC, and was offered as a staff meeting, an in-class demonstration, a parent presentation, a cooking class, or a staff mini-training.

### Outcomes

Each participating ECC was visited by two trained research assistants over two weekdays to collect data prior to the start of the intervention period and again 9 months later. This two-day data collection period was chosen for feasibility and logistical purposes, as well as to reduce the burden on ECCs. While blinding was not possible for the ECC, parents and children were not informed about group assignment. Research assistants responsible for collecting data were not told about the ECC’s group allocation.

#### Physical activity

Physical activity was assessed using the Actical accelerometer (B and Z-series, Mini Mitter/Respironics, Oregon, USA) [16], which has been shown to be a valid tool for measuring physical activity in preschoolers [17]. The Actical was worn by children during childcare hours for five consecutive days. Educators were instructed to place the accelerometer around each participating child’s waist when they first arrived at the ECC in the morning, and to remove it before the child went home at the end of the day. Once the measurement period of five work days was completed, the accelerometers were collected and sent back to the research team.

Accelerometer data were recorded in 15 s intervals. Time spent in physical activity (PA), moderate-to-vigorous PA (MVPA), light intensity PA (LPA) and sedentary time were measured based on predetermined validated thresholds for preschoolers [17]. Counts of less than 25 per 15 s represented sedentary time (which included nap time) [18], counts between 25 and 714 per 15 s represented LPA [17, 18] while counts of 715 and above defined MVPA [19]. Non-wear time was defined as any period of 60 consecutive minutes where no counts were measured. To provide the most reliable data while maximizing sample size, it was determined that children had to have worn the accelerometer for a minimum of 2 h on at least 4 days to be included in the analyses [19]. To control for within and between participant wear time variations, accelerometer data were standardized to an 8-h period, [20] which represents the typical number of hours children in our study attended the ECC. The SAS codes used to clean and manage raw accelerometer data for this study are available as open source [21].

#### Fundamental movement skills

The Test of Gross Motor Development (TGMD-II), a valid and reliable tool used to assess fundamental movement skills among children 3–11 years of age, was used to measure children’s fundamental movement skills [22]. Children were videotaped while completing two trials of each locomotor (run, hop, gallop, leap, horizontal jump) and object control skills (catch, kick, overhand and underhand throw), using the standard TGMD-II protocol. Videos were then reviewed by trained assessors who scored each skill and calculated a total raw score for locomotor skills and object control. The TGMD-II scoring protocol uses raw locomotor and object control skill scores to calculate an age adjusted Gross Motor Quotient (GMQ). The GMQ score applies a denominator which assumes that the child has performed each skill. However, some items of the TGMD were eliminated (slide, striking a stationary ball, and stationary dribble) due to the young age of the children. As a result the GMQ could not be accurately calculated for these children and thus, the Percent of Maximum Possible (POMP) scoring system was applied to score children’s fundamental movement skills [23]. The children’s raw object control and locomotor scores were converted to POMP scores to generate the maximum possible score based on the skills, which we included. This also enabled maximizing use of data for cases where children had missing data for a particular skill. For example, if a child had missing data for the run skill (i.e. because they did not want to run at the time of testing), the score for that child would be calculated on a maximum of 40 instead of having a score out of 48 as usual. POMP scores were computed, and age-adjusted as defined by the TGMD-II protocol.

#### Food intake and food served

Amount of food served by educators or cooks and children’s intake of vegetables and fruit (servings), fiber (g) and sodium (mg) were measured at lunch on 2 consecutive weekdays using weighed plate waste enhanced with digital photography. The intent of capturing at least two consecutive days of usual intake was to minimize the day by day variation in order to obtain a more representative measure while being logistically feasible. The weighed plate waste method has been shown to be a precise measurement of dietary intake [24, 25] and has been previously used in studies conducted among school-aged children [26–28]. This method consisted of weighing a standard serving of each food item served to the children. Digital photography was also used to document the weight of the food item sitting on the scale and its type or composition (e.g. mixed dish versus a single-ingredient item). Each child’s plate was weighed and photographed before each serving and after the child was done eating.. In the cases where children served themselves rather than being provided a pre-plated meal, each child’s individual servings of food were weighed and photographed in the same manner. If a second serving was requested by a child, the same procedure was repeated. With digital photography it was possible to estimate the quantity of individual food items first served and then left on each child’s plate. Food intake was calculated as the difference in weight between the total amount of food served and the amount of food leftover [25]. Plate waste data and recipes obtained from the childcare centres were used to assess the amount of vegetables and fruit, fibre and sodium served and consumed by each child, using the ESHA Food Processor nutritional analysis software, version 10.10.00 (Salem, Oregon). Finally, amount of vegetables and fruit (servings), fibre (g) and sodium (mg) served and children’s average intake over the 2 days of data collection were calculated.

#### Other variables

Children’s age and sex were obtained through a questionnaire administered to parents. The number of children in each ECC was based on the total number of preschoolers attending the centre. The ECCs were categorized as having 20 preschoolers or less, between 21 and 26 preschoolers or more than 26 preschoolers. The socioeconomic status of ECC was estimated based on the median income of individuals aged 15 years and older living within the same postal code as the ECC, using data from the Canadian 2011 National Household Survey [29]. Each ECC was placed into one of four socioeconomic status categories according to if their regional median income was less than $40,000, between $40,000 and $59,999, between $60,000 and $69,999, or $70,000 and above. As for geographical location, centres were defined as urban if they were in a census metropolitan area or a census agglomeration with a strong metropolitan influenced zone (MIZ), as defined by the Community Information Database, 2006 [15]. Centres were categorised as being in a rural area if they were in an area with moderate, weak or no MIZ.

Opportunities for physical activity and healthy eating were assessed using 55 items (25 items related to nutrition and 30 items related to physical activity) of the Nutrition and Physical Activity Self-Assessment for Child Care (NAP SACC) [30, 31] by two trained research assistants who scored the childcare centre’s environment over the two days of data collection. Each research assistant recorded their observations independently and compared their observations at the end of the second day. Excellent inter-rater reliability was shown between the research assistants (Cohen’s kappa = 0.942, *p* < 0.001). The mean ± SD for the scores of nutrition and physical activity components of the NAP SACC are reported separately for intervention and control groups at baseline (Table 1). The 55 items were summarized into fewer categories using principal component analysis. Given NAP SACC-derived variables were ordinal, we used the untie method (PRINQUAL procedure in SAS 9.4) to transform the data, which helps retain variance of the original data for finding correlations. The factor loadings are the correlation coefficient of the relationship between categories of the NAP SACC and the underlying factors [32]. For labelling the factors, we considered all questions with factor loadings above or below the cut off of ±0.4. Four groupings were identified to represent environmental factors related to physical activity and nutrition in ECC (see Additional file “1”).

**Table 1:** Descriptive characteristics of the study sample

	Control	Intervention
**CHILDREN LEVEL VARIABLES**	(*n* = 433)	(*n* = 462)
Age (years)	4.1 ± 0.75	4.1 ± 0.77
Sex (boys)	235 (54%)	237 (51%)
Height (cm)	102.8 ± 6.6	103.4 ± 6.6
Weight (kg)	17.1 ± 3.0	17.4 ± 3.1
Waist circumference (cm)	53.6 ± 4.6	53.6 ± 4.5
Age-adjusted BMI (kg/m2)	20.4 ± 3.7	20.3 ± 3.8
Province		
Saskatchewan	230 (53%)	272 (58%)
New Brunswick	203 (47%)	192 (42%)
Language of ECC		
English	265 (61%)	310 (67%)
French	168 (39%)	154 (33%)
Location of ECC		
Rural	152 (35%)	197 (42%)
Urban	281 (65%)	267 (58%)
Median household income (before taxes)	54,773 ± 10,790	54,769 ± 11,067
**CHILDCARE CENTRE LEVEL VARIABLES**	(*n* = 30)	(*n* = 31)
Number of children in ECC	27 ± 12	28 ± 15
Nutrition environment score (scale from 0 to 75)	38.31 ± 7.88	39.39 ± 7.23
PA environment score (scale from 0 to 90)	43.5 ± 10.27	42.0 ± 10.03

### Analyses

We used complete case analysis, such that only participants with complete outcome data were included. This represents a deviation from our original protocol, which planned for analyses to be pursued according to the intention-to-treat principle [14]. This modification was necessary as the issue of missing data largely affected outcome variables, and it is generally the norm not to use imputation for missing data among outcome variables, especially when the proportion of missing data is large [33]. To assess the effect of the intervention, measures of the outcomes of interest were fitted in mixed-effect models using time of measurement (baseline or endpoint), group (intervention or control), and an interaction between time and group as fixed effects (Models 1). Additional models (Models 2) were built on these initial models to account for potentially confounding variables identified using Directed Acyclic Graphs (DAG) for each outcome [34]. These graphs are frequently used in epidemiological studies as they help illustrate the potential sources of bias and help identify confounding variables which should be controlled in the statistical analyses [34]. DAGs help researchers visually represent their hypotheses and the relationships between the variables of interest. Specifically, 2 Models were adjusted for age, sex, size of ECC, neighbourhood income, language, province, rurality and a physical activity environment score for physical activity and gross motor skills outcomes, or a nutrition environmental score for food intake and food served outcomes. To account for clustering related to repeated measures and due to the sampling of participants in ECC, variables representing participants and ECC were included as random effects in all models. In a secondary set of analyses, we tested additional interaction terms to assess whether the intervention would have different effects across different strata (i.e., sex, province—Saskatchewan, New Brunswick, language of centre—English, French, or location of centre—urban, rural). Analyses were conducted with the MIXED procedure in SAS, version 9.4.

## Results

**Fig. 1:**
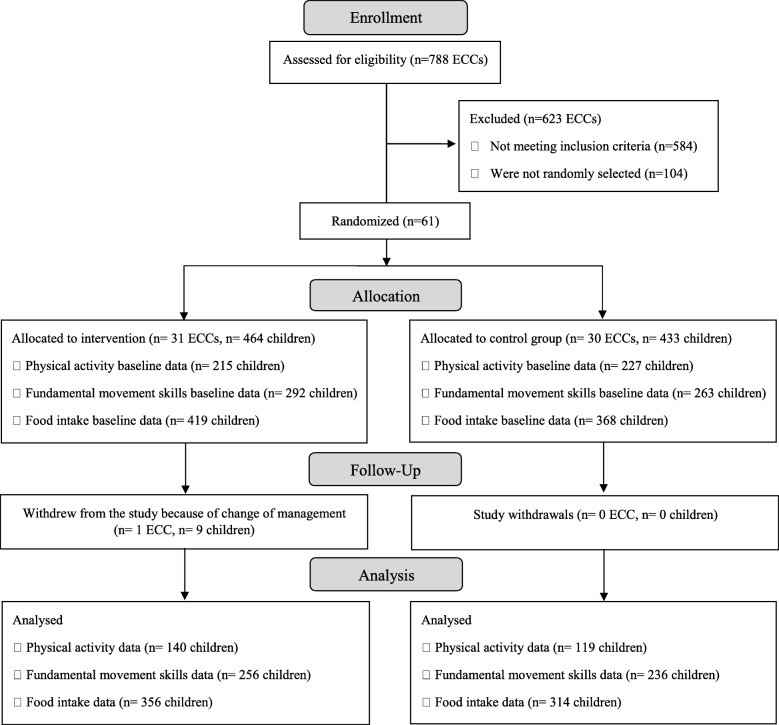
CONSORT flow diagram of participants through each stage of the intervention

Sixty-one childcare centres were randomly selected and allocated to the intervention or control group (Fig. 1). In total, 895 children (4.1 years old ±0.8) were recruited in September of 2013, 2014 and 2015. Of these children, 462 attended an ECC randomly allocated to the intervention group and 433 attended an ECC allocated to the control group. All ECCs allocated to the intervention group received the HSDS intervention, except for one centre (*n* = 9 participating children) which decided to drop out of the study due to change of management. Losses to follow-up were similar in both groups across all primary outcomes, except for physical activity where the percentage of follow-up loss was greater in the control group (25%) than in the intervention group (16%). The total number of children who provided valid data at both baseline and endpoint for PA, food intake and fundamental movement skills were 259, 670 and 492, respectively.

Following recruitment of one of the childcare centres in the usual practice arm, it was found that it had the same director and shared staff with a nearby ECC which had been recruited in the intervention arm. Given the risk of contamination quasi certain it was decided to amalgamate the 2 centres as one intervention centre.

Children in both groups were similar on all baseline characteristics as demonstrated in Table 1. On average, children lost to follow up engaged in more physical activities, displayed less sedentary time and had better scores on the object control component of the fundamental movement skills evaluation at baseline than children retained for the follow-up evaluation (Table 2).

**Table 2:** Baseline values of outcome variables among participants retained and lost to follow-up

	Participants Retained	Participants Lost to follow-up	*p*-value (t-test)
	Baseline values (mean ± standard deviation)		
**Physical activity**	(*n* = 259)	(*n* = 176)	
Total physical activity (minutes/day)	179.33 ± 44.41	189.98 ± 48.82	0.03
MVPA (minutes/day)	28.93 ± 15.34	32.11 ± 21.73	0.1
LPA (minutes/day)	150.41 ± 36.08	157.87 ± 38.53	0.06
Sedentary time (minutes/day)	300.67 ± 44.41	290.02 ± 48.82	0.03
**Fundamental movement skills**	(*n* = 492)	(*n* = 63)	
Locomotor (score)	41.6 ± 15.96	41.4 ± 16.09	0.9
Object Control (score)	42.04 ± 15.72	46.47 ± 14.95	0.05
**Food Intake**	(*n* = 670)	(*n* = 117)	
Fiber (g)	2.41 ± 1.34	2.39 ± 1.24	0.9
Vegetables and fruit (servings)	0.66 ± 0.43	0.61 ± 0.43	0.4
Sodium (mg)	502.48 ± 386.45	494.48 ± 324.32	0.9
**Food Served**	(*n* = 670)	(*n* = 117)	
Fiber (g)	2.84 ± 1.50	2.69 ± 1.35	0.4
Vegetables and fruit (servings)	0.80 ± 0.49	0.71 ± 0.47	0.2
Sodium (mg)	575.24 ± 415.94	553.92 ± 352.03	0.6

The models showed a positive effect of the intervention on locomotor skills (Table 3). Specifically, in the model controlling for potentially confounding variables, children in the intervention group had, on average, a 3.33 greater point increase in their locomotor motor skills scores than children in the control group. The intervention was not associated with statistically significant differences in effects on object control or physical activity variables. Overall, children in both the intervention and control group increased their time spent in total PA by approximately 10 min over an 8-h period, on average, between baseline and endpoint. Specifically, this represents an average increase of 7 min of MVPA and 3 min of LPA per childcare day. However, no significant differences in total PA, MVPA, LPA or sedentary activity were found between the intervention and control group between baseline and endpoint.

**Table 3:** Difference in PA, fundamental movement skills, food intake/served between the intervention and control groups^a^

	Control^b^ Group		Intervention^b^ Group		Models 1^c^		Models 2^d^	
	Mean (standard deviation) Pre	Mean (standard deviation) Post	Mean (standard deviation) Pre	Mean (standard deviation) Post	Beta for intervention-control group difference (standarderror)	*p*-value	Beta for intervention-control group difference (standarderror)	*p*-value
**Physical Activity**	(*n* = 119)		(*n* = 140)					
Total physical activity (minutes/day)	189.00 (42.23)	193.87 (44.12)	170.34 (44.70)	180.28 (48.11)	6.42 (6.18)	0.3	5.98 (6.28)	0.3
MVPA (minutes/day)	32.31 (16.98)	37.65 (18.36)	25.78 (12.97)	34.31 (17.65)	2.68 (2.49)	0.3	2.01 (2.54)	0.4
LPA (minutes/day)	156.69 (35.10)	156.22 (34.54)	144.56 (36.18)	145.98 (35.32)	3.81 (4.8)	0.4	4.11 (4.86)	0.4
Sedentary time (minutes/day)	291.00 (42.23)	286.13 (44.12)	309.66 (44.71)	299.72 (48.11)	−6.42 (6.18)	0.3	−5.98 (6.28)	0.3
**Fundamental movement skills**	(*n* = 236)		(*n* = 256)					
Locomotor (score)	44.35 (16.93)	44.72 (15.49)	38.55 (15.92)	43.02 (15.61)	3.84 (2.09)	0.001	3.33 (1.28)	0.009
Object Control (score)	45.41 (16.55)	43.69 (14.80)	43.02 (15.61)	44.08 (14.85)	3.38 (2.63)	0.201	1.61 (2.55)	0.5
**Food Intake**	(*n* = 314)		(*n* = 356)					
Fiber (g)	2.42 (1.42)	2.67 (1.74)	2.40 (1.44)	2.46 (1.37)	−0.09 (0.04)	0.05	−0.068 (0.047)	0.1
Vegetables and fruit (servings)	0.63 (0.52)	0.76 (0.69)	0.66 (0.46)	0.81 (0.57)	0.01 (0.03)	0.7	0.02 (0.03)	0.6
Sodium (mg)	474.94 (307.73)	485.79 (328.91)	528.92 (418.72)	521.07 (326.90)	−0.05 (0.7)	0.9	−0.12 (0.76)	0.9
**Food Served**	(*n* = 314)		(*n* = 356)					
Fiber (g)	2.84 (1.52)	2.99 (1.76)	2.68 (1.34)	2.73 (1.43)	−0.1 (0.04)	0.02	−0.07 (0.045)	0.1
Vegetables and fruit (servings)	0.76 (0.56)	0.85 (0.70)	0.76 (0.46)	0.92 (0.57)	0.04 (0.03)	0.2	0.06 (0.03)	0.05
Sodium (mg)	544.68 (336.68)	545.39 (348.24)	586.64 (430.55)	581.53 (387.46)	0.29 (0.7)	0.7	0.26 (0.73)	0.7

^a^Between group differences are based on mixed-effect linear regression models which include variables representing participants and childcare centres as random effects to account for repeated measures and for clustering of participants in childcare centres

^b^Means in this column are based on all data available at each measurement period

^c^Represents the interaction term between time and group. To be included in this analysis, participants had to have provided data for both the pre and post measurement periods

^d^Represents the interaction term between time and group with adjustments for age, sex, size of childcare centre, neighbourhood income, language, province, rurality and a physical activity environment score for physical activity and gross motor skills outcomes or a nutrition environmental score for food intake and food served outcomes

Whereas the intervention was not associated with differences in children’s food intake, the models suggested a marginal difference in food served following the intervention. Specifically, the adjusted model suggested a larger increase in portions of vegetables and fruits served in the intervention group compared to control group.

None of the interaction analyses suggested any difference in the effect of the intervention across sexes, provinces, language groups, or location of centres (data not shown).

## Discussion

The effectiveness of the HSDS intervention in increasing physical activity levels and healthy eating as well as improving fundamental movement skills in preschoolers attending ECC was only partially demonstrated. The intervention was associated with a statistically significant improvement in locomotor skills. This finding is not surprising as a process evaluation of the HSDS demonstrated that most ECC who received the HSDS intervention reported using the physical literacy HOP resource on a weekly basis and that 74% of those activities emphasized locomotor skills over object control skills [35]. Our findings are consistent with previous studies which have found that physical activity interventions targeting gross motor skills, result in an increase in locomotor skills but not necessarily object control skills [36, 37]. Interventions designed to increase gross motor skills in children often target locomotor skills such as jumping and running rather than object control skills. Wang et al. discussed this phenomenon and suggested that more targeted approaches should be employed when designing interventions aimed at supporting children in developing and improving both locomotor and object control skills [38].

The development of fundamental movement skills (locomotor, object control) in childhood are essential building blocks for participation in physical activity across the lifespan [36, 39]. Teaching children these essential movement skills may lead to a greater willingness to participate in physical activity of all types during early childhood and beyond [40]. Nevertheless, physical activity-level related outcomes were not associated with between group differences in this study. However, physical activity levels did improve in both groups. This positive change could potentially be indicative of a study effect (“Hawthorne effect”) in that children who wore accelerometers, regardless of intervention/control groups, were more active compared to when they were not wearing the devices. This observation is confirmed in 30% of the studies included in Waters et al. systematic review of control groups’ improvements in physical activity intervention trials and one of the associated factors was repeated measures [41]. Other possible explanations are the potential for seasonal effects as physical activity levels of preschoolers tend to increase during Spring [42, 43].

In terms of food intake, results did not reach statistical significance after controlling for confounding factors. According to a systematic review by Golley & Bell, previous interventions which provided nutrition training for ECC staff have found positive effects on children’s dietary intake or on centre food provision [44]. However, few studies showed a positive effect when these outcomes were assessed using objective methods [45, 46], as was done in this study. Overall, children’s food intake increased slightly in both groups. This could be attributed to a maturation effect, as children’s daily energy requirements increase by approximately 100 kcals each year between the age of 2 and 5 [47]. Furthermore beside the marginal increase in portions of fruits and vegetables served in the intervention group compared to the control group, no significant between-group differences in fiber, and sodium were found. The ECCs’ environment in which food is prepared and served, is influenced by provincial standards. Yet, the implementation of these standards in ECCs is limited by the lack of enforcement, [48] and their interpretation may vary as a function of the presence of a dedicated cook, access to fresh and affordable healthy food, and other contextual factors such as child care leadership and priorities, which are difficult to standardize, thus possibly explaining the modest direct impact on children’s diet.

As a population health intervention, HSDS’s main target was the change in the childcare centre’s environment with the hypothesis that in comparison to the usual practice group, exposure to the HSDS intervention would result in increased opportunities for physical activity and healthy eating in those centres which in turn would increase healthy behaviours in children. Impact on children hinged on the full deployment of the intervention as intended. According to our pilot study [49] and the HSDS process evaluation intervention, [35] educators were very responsive to HSDS, felt more confident in their own skills after the intervention training as well as were willing to organize the childcare centres environment and role model in order to facilitate behaviour changes in children. Reported changes were usually simple to implement, low cost and at the centre level. Educators’ modelling behaviours, skill development and increased self-efficacy are recognized in the literature as key strategies to effect change [49, 50] and our previously reported findings certainly concur with these. While implementation fidelity of the intervention was high, process evaluation results also showed that more ECC used the physical activity resource than the healthy eating resource. This could partially explain the dismal findings with regards to food intake and food served. Further, ECC generally reported lack of time, lack of support from childcare staff and low parental engagement as key challenges to full implementation and sustainability of HSDS, which could also explain the lack of significant results of this study. In Nixon’s systematic review of interventions in childcare settings targeting healthy behaviours and obesity prevention [10], 6 out of 12 studies documenting a significant improvement in outcomes were associated with medium to high parental involvement. This level of engagement was missing in the intervention reported here, therefore future interventions should more systematically target the whole family in addition to the ECC. Another variable to consider is the length of deployment in centres; maybe the intervention was too short or not sufficiently intensive.

The robust design of the study using a control group and pre and post objectives measures is a core strength of the HSDS study. Although applying the randomisation scheme, not all centres started at the same point at baseline and this situation may have overshadowed the true impact of HSDS, which was a population health intervention operating in real world settings, meaning that children and ECC in the usual practice group knew the purpose of the study in order to consent and were aware they would only receive the intervention in a delayed fashion in the future. The most obvious and visible measurements were related to the wearing of accelerometers, which may explain the increase in physical activity in both groups.

The lack of significant results could also be attributed to the following factors. It is possible that our priori statistical power estimates following pilot work were based on an optimistic level of 10% difference, which was not reached. Estimates may not have taken into consideration adequate sample size for achieving meaningful sub-groups analyses. Although the number of enrolled children surpassed our initial target number, the lower than anticipated number of participants who provided valid data at both time points, especially for physical activity, represents a limitation of the study which may have prevented us from demonstrating significant effects. In addition to reducing our statistical power, the loss of participants during follow up may have affected internal and external validity of our results. In particular, children who contributed to the outcome measures at both time points were generally less physically active and had poorer object control motor skills at baseline. Valid pre- and post-food intake data was easier to collect and control, as lunchtime was a structured and routine activity in the ECC, and therefore expected by children. The large proportion of missing data for the outcome variables also precluded the adoption of the intention-to-treat principle. It has been documented that deviating from the intention-to-treat principle may yield bias estimates, especially in cases where it is replaced by a per-protocol analysis [51]. In the current study, we used complete case analyses, which qualifies as a modified intent-to-treat approach where all participants for whom data were available were included, regardless of if their exposure occurred as planned in the protocol [52]. Although not as susceptible to bias as a per-protocol analysis, the complete case analyses used are associated with a higher risk that the study groups being compared differ in terms of potentially confounding variables than if the intention-to-treat principle were used [53]. Another limitation may be due to the length of the intervention, which was shorter than in studies reporting significant behaviours changes [54–56].

## Conclusions

In summary, HSDS, a population health intervention in the ECC settings combines increased opportunities for physical activity and healthy eating, which constitute two core components for effective childhood obesity prevention according to Bleich et al. recent systematic review [57]. However, although many (*n* = 12) outcome indicators were investigated, only locomotor skills at the child level and vegetables and fruits servings at the centre  level significantly increased at follow up, suggesting that the HSDS intervention was effective in only promoting some healthy behaviours among preschoolers attending ECC. No difference between the intervention and the control group were noted for the other variables assessed.

## Supplementary information

**Additional file 1.** Principal component analysis of the NAPSACC questionnaire.

## Data Availability

The datasets used and/or analysed during the current study are available from the corresponding author on reasonable request.

## Abbreviations

BMI: Body mass index
DAG: Directed Acyclic Graphs
ECC: Early childhood centres
GMQ: Gross Motor Quotient
HSDS: Healthy Start-Départ Santé
LPA: Light intensity physical activity
MVPA: Moderate-to-vigorous physical activity
NAP SACC: Nutrition and Physical Activity Self-Assessment for Child Care
PA: Physical activity
POMP: Percent of Maximum Possible
SD: Standard deviation
TGMD-II: Test of Gross Motor Development II
