# Supplementary material for: Effectiveness of the Healthy Start-Départ Santé approach on physical activity, healthy eating and fundamental movement skills of preschoolers attending childcare centres: a randomized controlled trial
Source: BMC Public Health. 2020 Apr 19;20:523. doi: 10.1186/s12889-020-08621-9 (PMC7169026; doi:10.1186/s12889-020-08621-9)
Supplement: Supplementary file 1 — Additional file 1. Principal component analysis of the NAPSACC questionnaire. [file 12889_2020_8621_MOESM1_ESM.pdf]

NAP SACC	Before intervention factors: Name and questions with positive or negative loadings on the factor	Factor loading corresponding to the NAP SAAC questions included in the questions
**Nutrition**	Factor 1. *"Improvement required on feeding environment and practices, education and professional development and policy"*.	
	+ Loading: Q2, Q5, Q7, Q8, Q9, Q11, Q12, Q13, Q14, Q19, Q20	+Loading: 0.85,0.99,0.99,0.99,0.99,0.99,0.99,0.99, 0.99,0.99,0.99
	- Loading: Q4,Q6,Q15,Q23,Q24,Q25	- Loading: -0.99,-0.99,-0.42,-0.87,-0.75,-0.99
	Factor 2 *"Menus, variety and educational and professional development"*	
	+Loading: Q1,Q2,Q10,Q15,Q18,Q21,Q22,Q23, Q24	+ Factor loadings: 0.91,0.48,0.79,0.85,0.95,0.99,0.98,0.48,0.6 5
	- Loading: Q16,Q17	- Factor loadings: -0.94,-0.77
**Physical activity**	Factor 3. "Beneficial Teacher Practices, Outdoor Play Environments and weak Professional Development"	
	+ Loadings: Q26,Q29,Q33,Q34,Q35,Q36,Q45,Q46, Q47,Q48,Q51,Q52,Q53,Q54,Q55	+Loading factors: 0.99,0.62,0.99,0.99,0.99,0.99,0.83, 0.99,0.99,0.99,0.99,0.99,0.99,0.99,0.99,
	- Loadings: Q28,Q38,Q39,Q49	- Loading factors: -0.99,-0.99,-0.95,-0.99
	Factor 4 "Beneficial Indoor Play Environment, less Outdoor Playtime"	
	+Loading: Q27,Q29,Q30,Q31,Q37,Q45,Q50	+ Factor loading: 0.75,0.77,0.98,0.97,0.93,0.54,0.40
	-Loading: Q32,Q43,Q44	-Factor loadings: -0.99,-0.85,-0.83

Additional file 1: Principal component analysis of the NAP SACC variables
